# Typhoon-related Leptospirosis and Melioidosis, Taiwan, 2009

**DOI:** 10.3201/eid1707.101050

**Published:** 2011-07

**Authors:** Hsun-Pi Su, Ta-Chien Chan, Chao-Chin Chang

**Affiliations:** Author affiliations: Centers for Disease Control–Department of Health, Taipei, Taiwan (H.-P. Su);; China Medical University, Taichung, Taiwan (H.-P. Su);; Kaohsiung Medical University, Kaohsiung, Taiwan (H.-P. Su);; National Taiwan University, Taipei (T.C.-Chan);; National Chung Hsing University, Taichung (C.-C. Chang)

**Keywords:** leptospirosis, melioidosis, natural disaster, typhoon, bacteria, zoonoses, letter

**To the Editor:** Global climatic changes have resulted in more natural disasters worldwide. These natural disasters can then cause outbreaks of emerging infectious diseases, including leptospirosis and melioidosis (*1**–**7*). In 2009, the moderate-strength Typhoon Morakot, with a maximum cumulative rainfall amount up to 3,059.5 mm, damaged Taiwan. After this natural disaster, unusual epidemics of leptospirosis and melioidosis occurred. The main objective of this study was to clarify whether these epidemics have resulted from this natural disaster.

Information about past typhoons that affected Taiwan was collected from the website of the Taiwan Meteorological Bureau (http://photino.cwb.gov.tw/tyweb/mainpage.htm; www.cwb.gov.tw) during January–August, 2009. The influential period of Morakot was in the 32nd week (August 5–August 10) in 2009. To evaluate the effects of this specific natural disaster, we divided the period into 2 intervals for analysis. The early period (before the typhoon) was from the 28th through the 32nd weeks, and the latter period (after the typhoon) was from the 33th through the 37th weeks in 2009. Information regarding 16 typhoons from 2000 through 2009 was further collected to evaluate effects of typhoon level, rainfall level, and maximum cumulative rainfall amounts on case numbers of leptospirosis and melioidosis after a typhoon.

The historical records of numbers of leptospirosis and melioidosis cases for analysis were obtained from the database collected weekly by the Centers for Disease Control, Taiwan. The information was referred to the website of the Taiwan Center for Disease Control (http://nidss.cdc.gov.tw/). To assess geographic variations, the age-adjusted incidence rates per 100,000 persons of leptospirosis and melioidosis were calculated in each city and county in Taiwan from February through September 2000–2009. SPSS version 15.0.0 software (SPSS Inc., Chicago, IL, USA), ArcGIS (ArcMap, version 9.3; ESRI Inc., Redlands, CA, USA), and SaTScan version 8.0 (www.satscan.org) were used for statistical analysis.

As shown by Mann-Whitney U test, frequencies of leptospirosis and melioidosis cases before the typhoon were significantly lower than those after the typhoon (all p<0.05). Furthermore, more leptospirosis and melioidosis cases were observed during the posttyphoon period in 2009 than during 2006–2008 (all p<0.05) (Figure).

**Figure:**
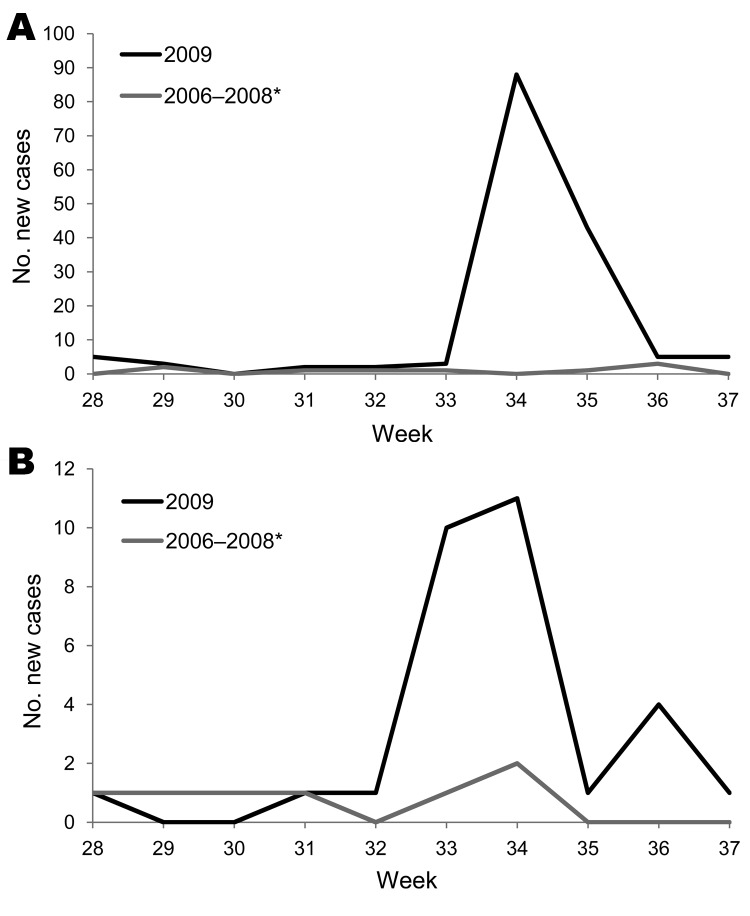
Comparison of epidemic curves during 2009 and 2006–2008. A) Epidemic curves of leptospirosis. B) Epidemic curves of melioidosis. 2006–2008* indicates that the curve was made by plotting the average weekly numbers.

Using Pearson correlation test to evaluate the effect of cumulative rainfall from Morakot, we found a positive correlation for leptospirosis (r = 0.54, p<0.05) and for melioidosis (r = 0.52, p<0.05). Effects of typhoons on numbers of leptospirosis and melioidosis cases in the late stage of a typhoon were further analyzed by using records of typhoon level and maximum 24-hour cumulative rainfall during 2000–2009. After weighting typhoon levels with scores (strong typhoon: 5 points; moderate typhoon: 3 points; mild typhoon: 1 point), we found that typhoon level with higher weight was significantly correlated with more cases of leptospirosis and melioidosis (r = 0.81 and 0.87, respectively; all p<0.05). The results further suggested that, when the 24-hour cumulative rainfall was >500 mm, significantly more meliodosis cases were observed (p<0.05). Although not statistically significant, the number of leptospirosis cases was positively correlated with 24-hour cumulative rainfall (r = 0.71; p = 0.14).

Using the Anselin local Moran statistic to evaluate geographic variations of leptospirosis and melioidosis after Morakot, we identified significantly higher incidence rates of melioidosis in Tainan, Kaohsiung, and Pingtung Counties in southern Taiwan (p<0.01). Nevertheless, no melioidosis cases were observed in Taitung, the county in the same latitude (20°N) but in eastern Taiwan. No significant geographic variation was found in the occurrence of leptospirosis. However, a high incidence of leptospirosis was observed in Pingtung, where flooding caused by Morakot was most serious (maximum cumulative rainfall >2,500 mm).

This study found that epidemics of leptospirosis and melioidosis possibly resulted from the moderate Typhoon Morakot. The findings implied that the effect of typhoon strength on the case numbers of leptospirosis and melioidosis could be less than that of rainfall level and maximum cumulative rainfall amount. Of major importance, the number of melioidosis cases was positively correlated with rainfall level >500 mm. The study further indicated that typhoon strength level and total amount of rainfall must be studied separately to determine their effects on epidemics of infectious diseases. The current typhoon classification system is only related to its intensity, which might not be always associated with total rainfall. The results of this study also implied that epidemic of melioidosis was more likely to be restricted to some geographic regions; this finding was not observed for epidemics of leptospirosis.

In conclusion, this study suggests that natural disasters, such as typhoons, that engender large amounts of rainfall could result in epidemics of leptospirosis and melioidosis. More in-depth studies need to be conducted. Efforts need to be taken in advance to prevent possible transmission of these infectious diseases after typhoons.
